# Examining the Influence of Anion Nucleophilicity on the Polymerisation Initiation Mechanism of Phenyl Glycidyl Ether

**DOI:** 10.3390/polym11040657

**Published:** 2019-04-10

**Authors:** Fiona C. Binks, Gabriel Cavalli, Michael Henningsen, Brendan J. Howlin, Ian Hamerton

**Affiliations:** 1 Department of Chemistry, Faculty of Engineering and Physical Sciences, University of Surrey, Guildford GU2 7XH, UK; fiona.c.binks@gmail.com (F.C.B.); g.cavalli@qmul.ac.uk (G.C.); b.howlin@surrey.ac.uk (B.J.H.); 2 Intermediates Division, BASF AG, Carl-Bosch-Straße 38, 67056 Ludwigshafen, Germany; michael.henningsen@basf.com; 3 Bristol Composites Institute (ACCIS), Department of Aerospace Engineering, School of Civil, Aerospace, and Mechanical Engineering, Queen’s Building, University of Bristol, University Walk, Bristol BS8 1TR, UK

**Keywords:** epoxy resins, phenyl glycidyl ether, ionic liquids, initiators, latent cure

## Abstract

The reaction of phenyl glycidyl ether (PGE) with 1-ethyl-3-methylimidazolium acetate and 1-ethyl-3-methylimidazolium thiocyanate to initiate the polyetherification reaction was examined using thermal and spectral analysis techniques. The influence of the nucleophilicity of the anions on the deprotonation of the 1-ethyl-3-methylimidazolium cation determined the reaction pathway. The thermal degradation of the ionic liquid liberated the acetate ion and led, subsequently, to the deprotonation of the acidic proton in the imidazole ring. Thus, polymerisation of PGE occurred via a carbene intermediate. The more nucleophilic thiocyanate anion was not sufficiently basic to deprotonate the 1-ethyl-3-methylimidazolium cation, and thus proceeded through direct reaction with the PGE, unless the temperature was elevated and a competing carbene mechanism ensued.

## 1. Introduction

Imidazoles are very effective initiators for the polymerisation of epoxy resins [1,2] as they catalyse the homopolymerisation of the highly-strained oxirane (epoxide) group. This reaction occurs easily at comparatively low temperatures to produce polyethers that are less highly crosslinked and, thus, more resilient [3] than some of their polyamine-cured counterparts [4]. Imidazole-cured epoxy resins display a variety of attractive thermal, mechanical, and electrical properties [5,6,7,8,9]. Consequently, these initiators have attracted interest from commercial producers of adhesives and anti-corrosion coatings [10,11]. However, the simple nature of the initiation mechanism leads to comparatively poor shelf life. Various strategies have been explored to improve the storage stability of imidazoles [12]. An alternative approach to improve storage stability involves the use of imidazolium-based ionic liquids (commonly called 1,3-dialkylimidazolium species), which are structurally related to imidazoles, but are characterized by stability and low viscosity [13], and are capable of rapid, high yielding polymerization reactions. The ease of synthesis [14] means that a range of potential structures is available, with particular emphasis on the substitution of different alkyl residues in the 1-position of the heterocyclic ring to modify solubility (e.g., ethyl [15], butyl [16], or decyl residues [17]), where the 3-position is typically substituted with a methyl residue. The nature of the counter-ion (e.g., chloride, tetrafluoroborate, or dicyanamide [17]) generally influences the stability of the ion pair, thus affecting the longer-term storage stability. Consequently, the inclusion of a tetrafluoroborate ion [16] yielded a commercial epoxy (Epidan 6)-ionic liquid formulation capable of storage at ambient temperature for six months. The introduction of dicyanamide [15] produced a miscible, stable blend in Epon 828 epoxy, which exhibited latent polymerisation behaviour through the reaction of the anion with the epoxy. We have previously examined the nature of the initiation of diglycidyl ether of bisphenol A (DGEBA) using selected ionic liquids [18], but the difunctional nature of the monomer somewhat limited the range of conversion over which spectral data may be acquired. The current work examines the relationship between the initiation mechanism and the nucleophilicity of the ionic liquid through the use of a model epoxide, phenyl glycidyl ether, in a detailed spectroscopic study.

## 2. Experimental

### 2.1. Materials

1-ethyl-3-methylimidazolium dicyandiamide and 1-ethyl-3-methylimidazolium thiocyanate were supplied by BASF, and phenyl glycidyl ether (PGE, 99%) was purchased from Sigma Aldrich (now Merck, St. Louis, MO, USA). The materials were characterised using ^1^H nuclear magnetic resonance (NMR) spectroscopy and used without further purification (see Supplementary data, Figures S1 and S2).

### 2.2. Instrumentation

^1^H NMR spectra, comprising 16 scans, were conducted on a Bruker 300 MHz NMR spectrometer (Billerica, MA, USA) at 298 K. The samples for analysis (80 mg) were mixed with DMSO-*d*_6_ (0.6–0.7 mL) and transferred to an NMR tube. PGE (2 g) and the ionic liquids, 1-ethyl-3-methylimidazolium acetate (2.27 g) and 1-ethyl-3-methylimidazolium thiocyanate (2.25 g), were mixed and stored in a closed glass scintillation vial at room temperature. An aliquot (70–80 mg) was taken from the formulation at set intervals, mixed with deuterated acetone (80 mL), and placed in an NMR tube for analysis.

Raman spectra were recorded using a Perkin-Elmer System 2000 FT-NIR spectrometer (Waltham, MA, USA), which was initially set up for use in near infrared mode and was calibrated with sulphur for use as a Raman spectrometer, manufacturer, city, state, country. The laser power was optimized to achieve the lowest signal-to-noise ratio for each sample and 16 scans were recorded. Samples were contained in clear glass scintillation vials, which allowed the sample to be analysed directly without further preparation. The software used for the analysis of the data was Spectrum v 5.3.1. PGE (5 g). Each ionic liquid (0.25 g) was mixed in a clear glass scintillation vial and analysed every 5 min at a laser power of 150 mW in the region of 400–4000 cm^−1^, with 16 scans performed on each sample.

Differential scanning calorimetry (DSC) was undertaken using a TA Instruments Q1000 (New Castle, DE, USA), running the TA Q Series Advantage software, on samples (5.0 ± 0.5 mg) in hermetically sealed aluminium pans. Experiments were conducted at a heating rate of 10 K/min from 20 to 140 °C (heat/cool/heat) under flowing nitrogen (50 cm^3^/min).

For the room temperature exposure (discussed in Section 3.2), a sample of 1-ethyl-3-methylimidazolium acetate was dried under vacuum at 40 °C for approximately 15 h. The sample was subsequently divided between four glass scintillation vials that had been stored in a drying oven for 24 h prior to use and left without a lid on in the fume cupboard. The samples were weighed after 2, 3, and 6 days, and then combined with PGE for DSC analysis. Samples of the ionic liquid (0.05 g) after the various exposure times were mixed with PGE (1 g) and analysed using DSC at a scan rate of 10 K/min from 20 °C to 140 °C. The dried initiator was also analysed to confirm that the drying process had not affected the initiating ability of the material.

For the room temperature NMR study, a sample of the PGE and each ionic liquid were mixed and analysed over a period of 90 min (the ionic liquid was not dried prior to analysis).

For the freezer study (discussed in Section 3.4), the formulation was stored in the freezer and an aliquot taken for analysis

## 3. Results and Discussion

### 3.1. Background to the Mechanistic Study

**Figure 1:**
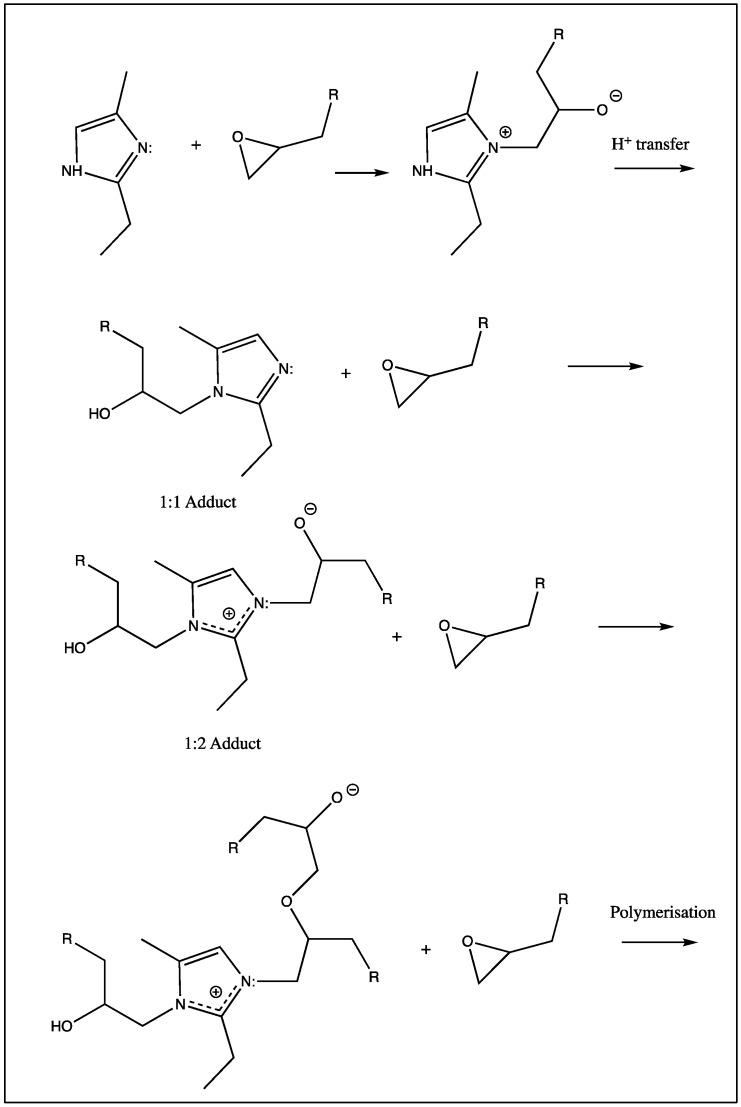
Mechanism of the reaction between 2-ethyl-4(5)-methylimidazole and phenyl glycidyl ether (PGE), as proposed by Barton and Shepherd (R = OPh) [20].

The groundwork for the mechanisms proposed for the initiation of epoxy resins using imidazoles and related species was laid in 1968 by Farkas and Ströhm [4], who studied the curing of PGE with 2-ethyl-4(5)-methylimidazole and suggested that the first stage of the reaction required the attack of the secondary N-3 atom on the terminal carbon atom of the epoxy group to generate a 1:1 adduct. They postulated that both nitrogen atoms on the imidazole ring participated in the curing process in the initial stages and become incorporated into the system [19]. Barton and Shepherd [20] revised this mechanism (Figure 1), having observed that the rate of reaction of the 1:1 adduct (formed from the imidazole and an epoxy) with another epoxide ring was of the same order as the rate of reaction of the original imidazole (2-ethyl-4(5)-methylimidazole) with epoxide.

This led to the conclusion that the N-1 nitrogen, as opposed to the N-3 nitrogen, was the reactive species. It was additionally noted that the rate of adduct formation appeared to be higher than the rate of polymerisation.

**Figure 2:**
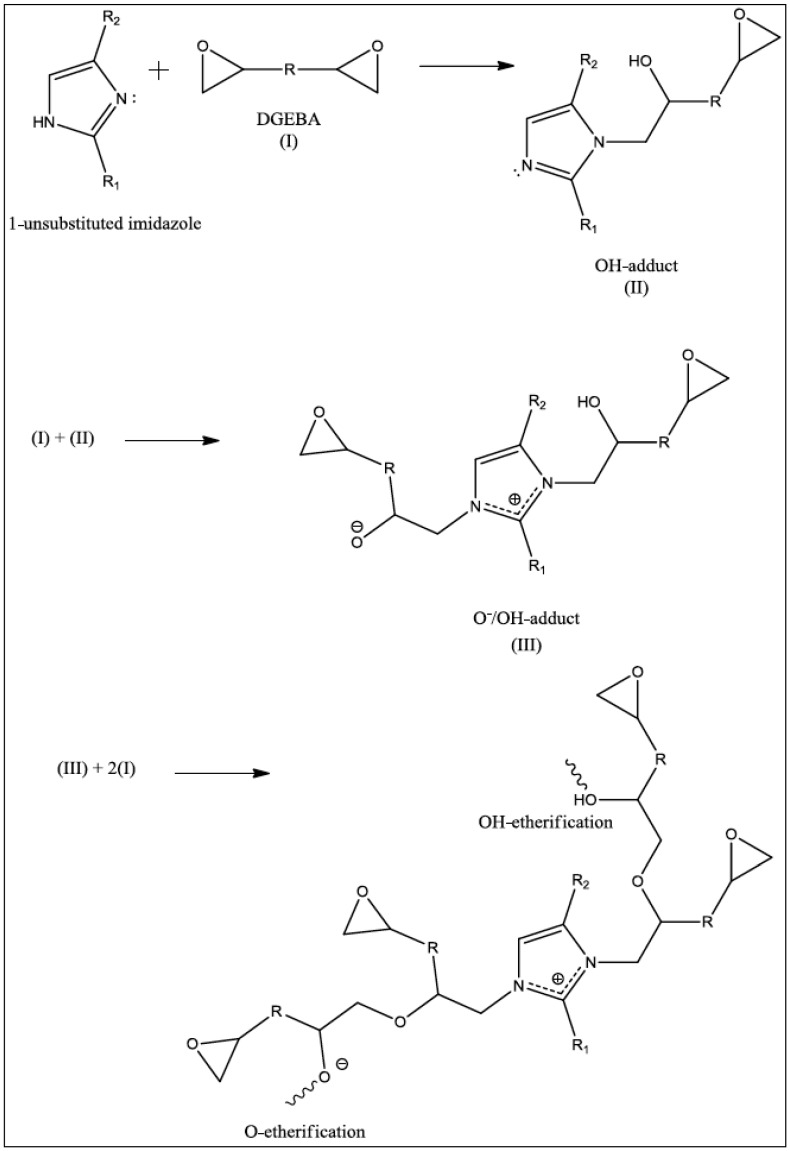
Mechanism of the reaction between 2-ethyl-4-methylimidazole and diglyidyl ether of bisphenol A (DGEBA) as proposed by Heise and Martin (R = CH_2_OPhC(CH_3_)_2_PhOCH_2_, R_1_, R_2_ = alkyl groups) [22].

Many studies have focused on epoxy/imidazole systems and, more specifically, on the reaction between PGE and various imidazoles [4,19,20,21]. It has been observed that the formation of epoxy/imidazole adducts appears to be the first stage in the curing process, as both nitrogen atoms are involved in the forming of adducts, with the imidazole becoming permanently incorporated into the network [4,20,22]. The N-1 and N-3 atoms are believed to act in the same manner as pyridine, and form both 1:1 and 2:1 epoxy/imidazole adducts through the ring opening of the epoxide group, with the 2:1 adduct thought to be the catalyst that initiates the polymerisation process [20,21,22]. Further studies on the reaction of the diglycidyl ether of bisphenol A (DGEBA) with low concentrations of 2-ethyl-4-methylimidazole resulted in a reaction mechanism, based on the previous work with PGE [20,21], being proposed. The proposed reaction mechanism, as shown in Figure 2, suggests that four different reactions are possible: two adduct reactions and two etherification reactions [22].

Heise and Martin [23] analysed the different reactions that occurred in DGEBA/imidazole systems and observed the effect they had on the thermal properties of the resulting network. The first adduct formation results from an attack of the 3-N atom of the imidazole on the epoxide group, causing it to open and form the 1:1 molar OH adduct through rearrangement of the zwitterion intermediate and a proton transfer. The second adduct is formed through an attack of the 1-N atom of the OH adduct on another epoxide ring to form the 2:1 molar O^−^/OH adduct. The latter, as mentioned previously, is believed to catalyse the polymerisation reaction due its highly reactive alkoxide ion. Owing to the fact that both adducts have similar rates of formation, it can be assumed that the N-1 atom is involved in both reactions rather than the hydrogen atom on N-3 reacting with the epoxide ring in a similar manner to an amine-curing agent, as was previously thought [4,20,21,22]. Previous work by the same authors had already established that adduct formation is necessary before the etherification reaction can proceed, and, hence, this is the rate-limiting step of the reaction [23]. Etherification can proceed via two different pathways, namely O-etherification or OH-etherification, and it is the chain growth via these two reaction points that allows for the cross-linking of the resin and the final network properties to be determined. It has been established that in order for both adducts to form, it is necessary to use a 1-unsubstituted imidazole in order to have a hydrogen atom present on the secondary nitrogen (N-3) atom that is capable of rearrangement during the zwitterion intermediate state. Therefore, in the case of e.g., 2-ethyl-4-methylimidazole, imidazole, and 1-unsubstituted imidazoles, both the OH (1:1) adduct and the O^−^/OH (2:1) adduct are formed, whereas for 1-substituted imidazoles, e.g., 1,2-dimethylimidazole and 1-methylimidazole, only the O^−^ (1:1) adduct is formed.

FTIR spectroscopy has been used to track epoxide concentration and, hence, to indirectly monitor the formation of the O^−^/OH adduct. The presence and subsequent disappearance of the N–H stretching peak in the FTIR spectrum allows for the formation of the OH-adduct to be directly monitored. In the case of 2-ethyl-4-methylimidazole, at a reaction temperature of 80 °C and an imidazole concentration of less than 25 mol %, the adduct formation reaction nears completion before the etherification reaction begins; there is no evidence of the formation of aliphatic ethers in the IR spectrum. Adduct formation for 1-unsubstituted imidazoles is characterised by a slow rate of epoxide conversion and a relatively constant glass transition temperature. The start of the etherification reaction is indicated by a rapid and sudden increase in the glass transition temperature, an increase in the rate of epoxide conversion, and the developing presence of a broad aliphatic ether band occurring in the IR spectrum in the region of 1140–1110 cm^−1^ [22]. FTIR spectroscopy data suggest that the adduct formation reaction approaches completion prior to the start of the etherification reaction. The etherification reaction is characterised by the presence of a second exothermic peak in an isothermal DSC thermogram. Thus, when comparatively higher initiator contents (e.g., 50 mol % imidazole) are used the adduct formation predominates and, consequently, consumes the majority of the epoxide groups, leading to a single exothermic peak. At 50 mol % or higher concentrations, the initiator is sufficiently present to cause a reaction of all of the epoxide groups to form adducts, the etherification reaction may begin earlier, and, in this case, the single exothermic peak will show a broad tail at the end rather than a separate exothermic peak. At intermediate imidazole concentrations (between 5 and 25 mol %), a second exothermic peak is clearly observed, and is attributed O-etherification, which increases as the concentration of imidazole decreases (i.e., fewer epoxide groups are available to form adducts).

Subsequent work by Ricciardi et al. [19] focused on the fact that differences in the catalytic ability of various imidazoles were observed, which is not accounted for in terms of the mechanism proposed by Barton and Shepherd [20]. Regardless of the imidazole, a similar adduct was formed with PGE (see Supplementary data Figure S3), yet it has been observed that the overall rate of reaction varies greatly depending on the nature of the imidazole [19]. Additionally, as the rate of adduct formation is faster than the rate of polymerisation, this is not this stage of the reaction that is rate-determining. The alkoxide anion, which was believed to be the reactive species in the etherification reactions, was shown to be less effective as a catalyst than the imidazole wherein the addition of alkoxide anions to the curing reaction mixture had little effect on the rate of the reaction. In contrast, the addition of an equivalent amount of an imidazole significantly increased the rate of the reaction. Consequently, the authors inferred that the imidazole curing agent was active throughout the polymerisation process rather than just at the start, during adduct formation, as was previously thought [19]. This was presumed to be the reactive species, however, when the authors attempted to prepare this adduct, a mixture of several compounds resulted. In most cases the methyl group on the imidazole was removed, and in only a few cases had this group been transferred to the hydroxyl group on the side chain. Additionally, some 2-substituted imidazoles were formed (see Supplementary data Figure S4).

In order to isolate the effect of the imidazole portion of the system’s reactivity from the PGE chain contribution, a 1,3-disubstituted imidazole (1,3-dimethylimidazolium iodide), displaying an increased electrophilic imidazole ring due to the quaternization process, was prepared and treated with various nucleophiles ranging in character from weak bases, such as the phenyl selenate anion, to strong bases, such as the methoxide ion, to observe the effects of nucleophilicity. Nuclear magnetic resonance (NMR) spectroscopy showed that in all cases the imidazolium ring remained intact, although N-demethylated product was also observed in each case. In the case of 1,3-dimethylimidazolium iodide, the cleavage of the N–C bond—essentially the reverse of the salt-forming reaction—resulted in the regeneration of 1-methylimidazole. It was observed that, upon heating the imidazolium salt to above 200 °C, demethylation occurred, yielding 1-methylimidazole and methyl iodide. Subsequent investigation showed that heating the corresponding hydroxide salt (1,3-dimethylimidazolium hydroxide) to above 100 °C resulted in 1-methylimidazole being regenerated.

**Figure 3:**
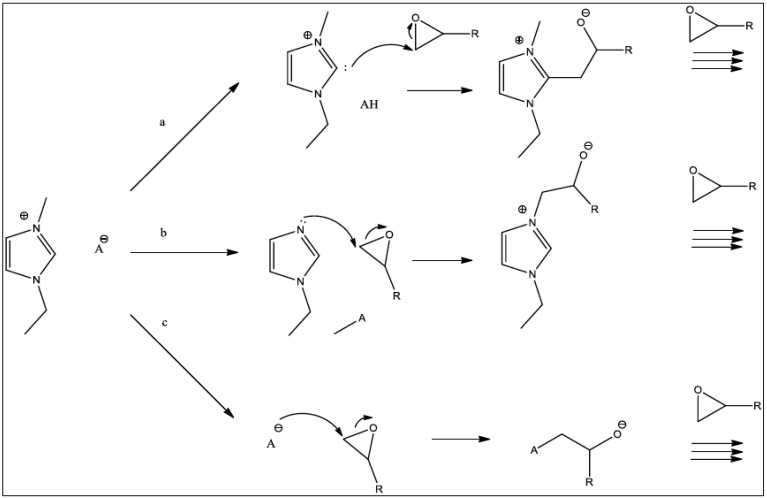
The proposed reaction pathways for the initiation of an epoxy polymerization reaction with imidazolium-based ionic liquids via (a) the “carbene route”, (b) the “imidazole route” and (c) the “counter ion” route.

The potential of ionic liquids as processible, easily-formulated, and stable initiators has already been mentioned, which makes them increasingly attractive in technological applications. The thermal decomposition of imidazolinium-based ionic liquids has been studied extensively, with authors generally concluding that a dealkylation mechanism occurs. Ohtani et al. [24] investigated a range of 1,3-dialkylated imidazolium-based ionic liquids and found that the thermal decomposition of the imidazolium-based ionic liquids occurs mainly through the cleavage of the C–N bonds within the chains (although C–C cleavage also occurs in longer alkyl chains). If a halide is present, then the corresponding haloalkanes and 1-alkylimidazoles are generated by nucleophilic attack of the halide on the alkyl residue. The anionic initiation mechanism is complex (Figure 3) and the thermal decomposition of the ionic liquid yields a highly stabilised *N*-heterocyclic carbon structure that may generate imidazole or 1-alkyl derivatives. Several routes are then possible: a “carbene route” (following proton abstraction from the carbon between the nitrogen atoms), an “imidazole route” (involving dealkylation followed by nucleophilic attack of the N-1 atom in the imidazole ring), and a “counter ion route”.

### 3.2. Examining the Storage Stability of PGE and 1-Ethyl-3-Methylimidazolium Acetate

**Figure 4:**
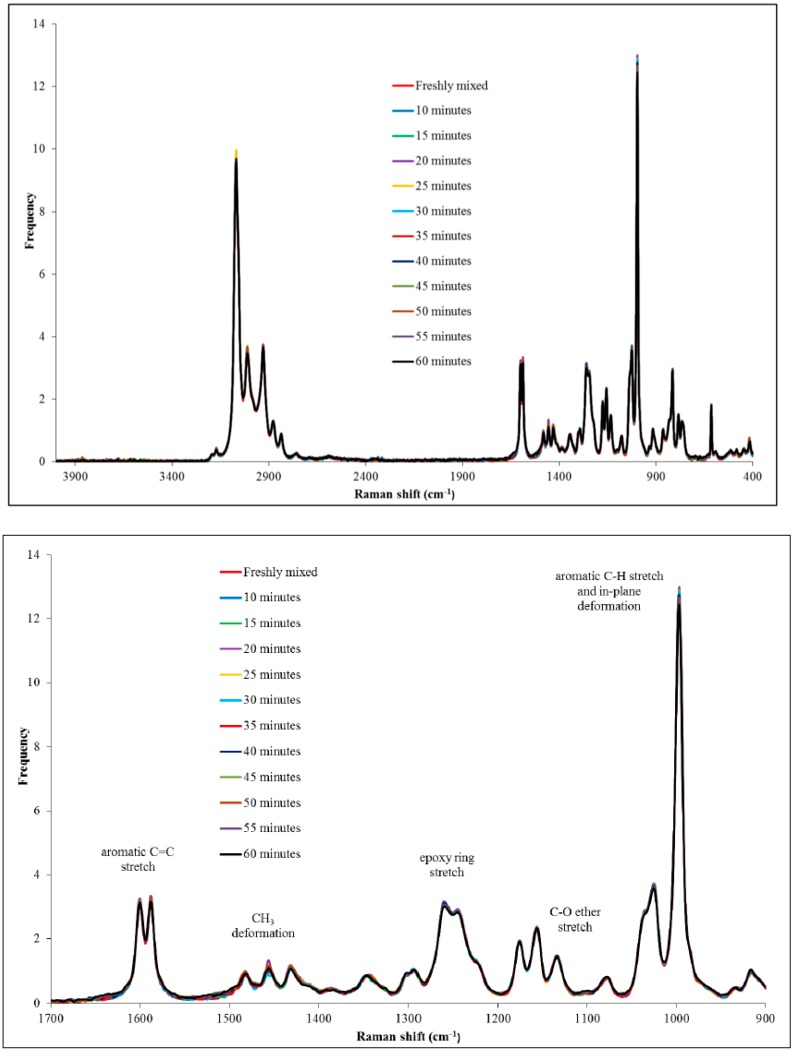
Raman spectra for a room-temperature-stored formulation comprising of PGE (5 g) and 1-ethyl-3-methylimidazolium acetate (0.25 g) as a function of time (**top:** full spectrum and **bottom:** expansion).

PGE was selected as the epoxy in the present investigation as there is a significant colour change, from light yellow to dark red (see Supplementary data Figure S5), which occurs within a short time period (60 min) when it is mixed with 1-ethyl-3-methylimidazolium acetate. Thus, PGE (5 g) and 1-ethyl-3- methylimidazolium acetate (0.25 g) were mixed in a clear glass scintillation vial and analysed every 5 min at a laser power of 150 mW in the region 400–4000 cm^−1^ (Figure 4).

It was apparent that no marked differences were observed between the spectra obtained at different times. During the 60 min analysis period, the sample was observed to turn from a very light yellow to a very dark red/brown, although no obvious increase in viscosity was discerned. In order to ascertain what occurred during this time frame, peaks of interest were studied, such as the epoxy ring stretch and the C–O ether stretch (Figure 4, bottom). No significant differences were observed between peaks, suggesting that the initial reaction, encompassed by the shoulder reaction, did not involve the epoxy ring to a significant degree.

**Figure 5:**
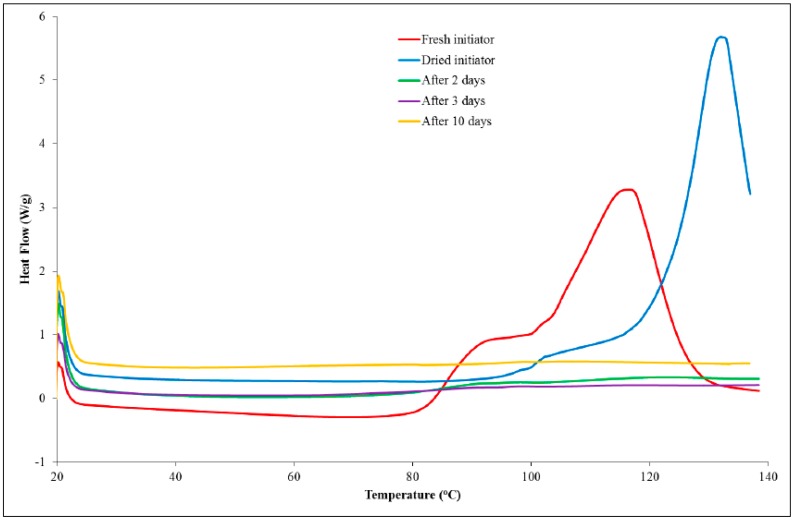
Dynamic DSC data for formulations comprising PGE (5 g) and dried or exposed 1-ethyl-3-methylimidazolium acetate (0.25 g).

Samples of a formulation, comprising PGE (1 g) and 1-ethyl-3-methylimidazolium acetate (0.05 g), were analysed using DSC at a scan rate of 10 K/min from 20 to 140 °C following different storage times. The dried initiator was also analysed using DSC to confirm that the drying process had not affected the initiating ability of the material. The DSC thermograms are shown in Figure 5. Upon addition of the ionic liquid to PGE, small globules were formed that remained after mixing, indicating that the two liquids were immiscible. The DSC data for the formulation of 1-ethyl-3-methylimidazolium acetate and PGE, stored at room temperature for a period of 60 min, revealed that a low temperature shoulder peak had indeed been lost or, at least, greatly reduced in size. Subsequent DSC analysis showed that the initiating ability of the ionic liquid had been lost as no exothermic peaks were observed in the thermograms. The dried sample, which had not been exposed to the atmosphere, was shown to delay the onset of the reaction considerably and to shift the T_max_ value to a higher temperature compared to the sample that was taken freshly from the bottle. The fact that the ionic liquid was shown not to initiate the reaction can be explained by the existence of hydrogen bonding and the proton abstraction effect in the material [19]. The ability of the dried sample to considerably delay the onset of the reaction may well be a result of the loss of water, which could catalyse the etherification mechanism in the later stages of the reaction. While the main etherification reaction (leading to the major DSC peak) results in changes in the peaks of interest in the Raman spectrum, it is likely that the first reaction does not involve a sufficient number of epoxy groups to cause either a decrease in the peak characteristic of the epoxy group or an increase in the peak, which is attributed to an ether stretch.

**Figure 6:**
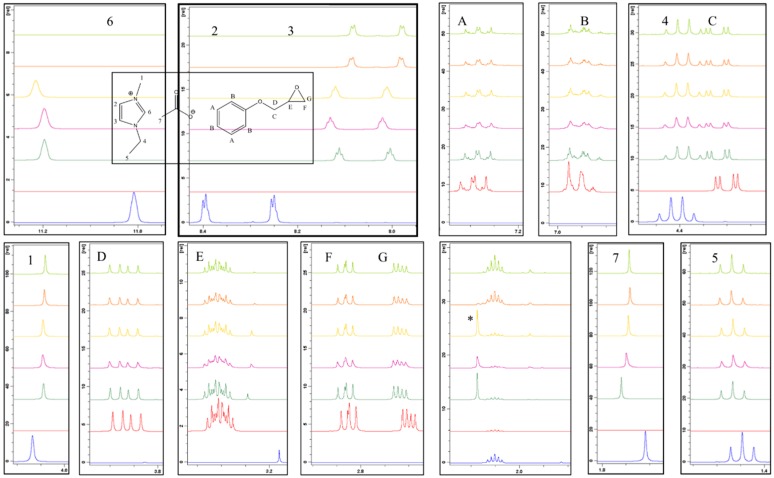
Expanded overlaid ^1^H NMR spectra of a room-temperature-stored 1:1 formulation of PGE and 1-ethyl-3-methylimidazolium acetate.

Complementary analysis, via NMR spectroscopy, allowed for more subtle changes in the formulation to be observed. A formulation based on an equimolar 1:1 stoichiometry was employed to highlight chemical changes although, in the absence of heat, this was not expected to impact the reaction mechanism significantly. The expanded overlaid ^1^H NMR spectra (Figure 6) included, from bottom to top, 1-ethyl-3-methylimidazolium acetate (dark blue), PGE (red), 0 min (dark green), 30 min (purple), 50 min (yellow), 70 min (orange), and 90 min (light green). Any new peaks, which cannot be assigned to the starting materials, are highlighted with an asterisk (*). In this case, a number of changes occurred over the storage period. The acidic proton (H_6_) shifted downfield upon the addition of the epoxy and greatly reduced in size until the point at which it was no longer observable in the spectrum without significant magnification. Integration of the peak area suggested that, in general, the peak decreased relative to the aromatic ring protons (H_A_), over the storage time, although the trend was not linear (Table 1). This suggests that the mechanism does not simply consist of one reaction and is likely to be influenced by other processes, which would account for the area of the peak to fluctuate.

**Figure 7:**
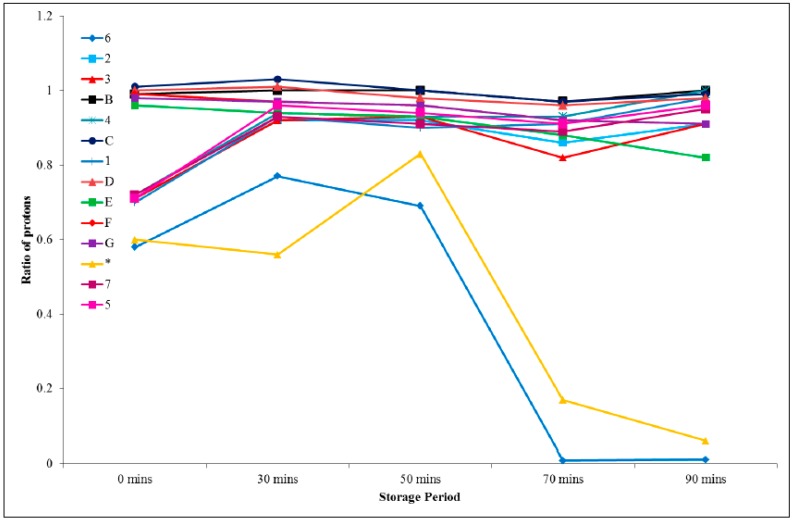
Ratio of proton integral change in a room-temperature-stored 1:1 formulation comprising PGE and 1-ethyl-3-methylimidazolium acetate.

Protons H_2_ and H_3_ on the imidazolium ring experienced a slight upfield shift upon the addition of the epoxy, however, they retained a fairly constant chemical shift over the storage period and integrated to the same value relative to the protons labelled as H_A_. This upfield shift is consistent with the deprotonation that occurs as the electron density of the ring increases, meaning that the protons on the ring and alkyl groups will be more shielded. Protons H_A_, H_B_, H_C_, H_D_, H_E_, H_F_, and H_G_, which are attributable to the PGE component, did not show significant changes in their chemical shifts when combined with the ionic liquid, however, integration of the peaks attributed to protons H_E_, H_F_, and H_G_, which are associated with the epoxy group, revealed a decrease in area over the storage period. The intensity of the aromatic ring protons (H_A_) should not change during the reaction, since these protons do not participate in the reaction mechanism. The ratio of the integral change for each proton was expressed with respect to H_A_ and the results are represented graphically in Figure 7. From this treatment, it can be seen that proton H_6_ exhibits the greatest change over time.

The ^13^C NMR spectra (not shown) revealed the presence of no new peaks, but showed that the peak attributed to the NCHN carbon was not visible in the spectrum after a period of 70 min of storage time. This supports the ^1^H NMR spectral data that suggest the suppression of the NCHN proton after the same storage period.

### 3.3. Examining the Storage Stability of PGE and 1-Ethyl-3-Methylimidazolium Thiocyanate

**Figure 8:**
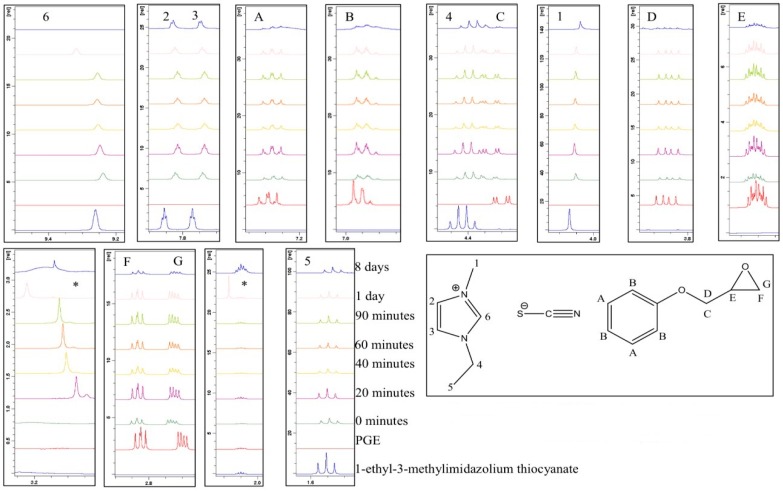
Expanded overlaid ^1^H NMR spectra of a room-temperature-stored 1:1 formulation of PGE and 1-ethyl-3-methylimidazolium thiocyanate.

**Figure 9:**
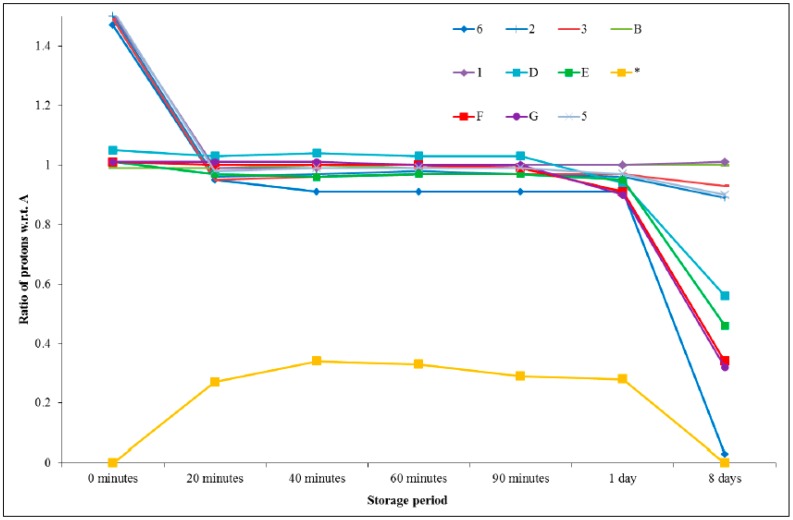
Changes in the ratio of proton integrals during room temperature storage for a 1:1 formulation comprising PGE and 1-ethyl-3-methylimidazolium thiocyanate.

A similar analysis was performed on a formulation comprising PGE and 1-ethyl-3-methylimidazolium thiocyanate. Maka et al. had previously studied a similar ionic liquid in the context of epoxy cure (with DGEBA) [17]. They reported that the initiation of the epoxy polymerisation with 1-butyl-3-methylimidazolium thiocyanate proceeded via the aforementioned thermal decomposition and yielded products that included imidazole and alkyl derivatives. In our work, the formulation changed colour markedly from light yellow to dark red over approximately 60 min at room temperature. The emergence of the new peak (*) occurred in the early stages and was attributed to the formation of an intermediate species as a result of the reaction between the thiocyanate anion and the epoxy ring. It was apparent that the chemical shifts remained very similar over the analysis period (Figure 8). Table 2 provides a summary of all the integrals and ratios for each peak with respect to the aromatic ring protons (H_A_), which were thought to remain constant throughout the analysis. These ratio data are presented graphically in Figure 9, from which it can be seen that the acidic proton (H_6_) remained fairly constant in its integral value after an initial decrease in the first 20 min until a period of one day was reached, and then diminished dramatically after 8 days. The protons on the epoxy ring, and in close proximity (H_D_, H_E_, H_F_, and H_G_), remained fairly constant for a period of 90 min and were then seen to begin decreasing (in terms of the integration value). The new peak which emerged after 20 min had a relatively constant value, with respect to the aromatic protons, but disappeared after 8 days. The remaining peaks did not vary significantly in intensity.

**Figure 10:**
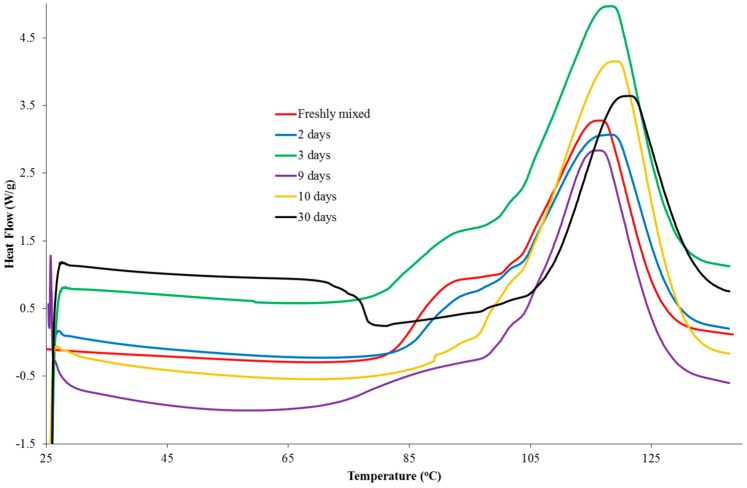
Comparison of the dynamic DSC data for freezer-stored formulations of PGE (5 g) and 1-ethyl-3-methylimidazolium acetate (0.25 g) as a function of storage time.

The fact that the chemical shift for proton H_6_ remained visible, and there was not such a marked downfield shift in the imidazolium ring protons, suggests that, at room temperature, the reaction between 1-ethyl-3-methylimidazolium thiocyanate and PGE does not proceed via a carbene initiation pathway. The appearance of a chemical shift at approximately 2.0 ppm (marked with an asterisk) in the spectrum acquired after one day is unidentified, although it is hypothesised that it may be due to residual acetone in the NMR tube after a cleaning process was employed and, as such, will be discounted from any discussion. Figure 8 shows the expanded overlaid ^1^H NMR spectra for a sample analysed over a period of 8 days. Any new peaks, which cannot be assigned to the starting materials, are highlighted with an asterisk (*). It can be seen from Figure 10 that the chemical shifts remained very similar over the analysis period. Table 2 provides a summary of all the integrals and ratios of each peak with respect to the aromatic ring (H_A_).

### 3.4. Thermal Analysis of Formulation Stored in the Freezer

The freezer study was repeated using PGE as the epoxy and 1-ethyl-3-methylimidazolium acetate as the initiator, with the aim of probing the disappearance of the low temperature shoulder reaction, which was more evident when PGE was used as the epoxy component of the formulation. Samples (0.5–0.7 mg) were prepared in the same manner as above and used for the analyses, which implemented a heat-cool-heat cycle from 25 to 140 °C at 10 K/min. Whilst the results (Figure 10) displayed some scatter, it was still possible to observe the loss of the first peak around 85 °C. In the freshly mixed sample there was a clear plateau region in the thermogram between approximately 90 and 99 °C, which could be seen in the two-, three-, and nine-day samples. This region was lost in the 10-day sample and an unusual drop in heat flow was seen in the 30-day sample, which may be indicative of a glass transition temperature that occured due to the advancement of the reaction during the storage period.

## 4. Conclusions

NMR spectroscopy revealed a significant difference in the initiation mechanisms involved in the polymerisation of PGE when initiated with 1-ethyl-3-methylimidazolium acetate and 1-ethyl-3-methylimidazolium thiocyanate. The presence of a chemical shift in the ^1^H NMR spectrum, corresponding to the acidic hydrogen in the imidazole ring, remained relatively unchanged in the thiocyanate formulation for a significantly longer period of time than for the acetate formulation. Whilst it appeared that deprotonation occurred when the epoxy was mixed with the ionic liquid containing the acetate counter ion, there was no evidence to suggest a similar process occurred in the case of the thiocyanate anion. This is likely due to the fact that the thiocyanate anion is not sufficiently basic to abstract the acidic proton. This finding is consistent with the DSC data, which showed that the onset of the reaction occurred at a lower temperature when 1-ethyl-3-methylimidazolium acetate was used in the formulation. When the nucleophilicity of the anions is considered, it is apparent that the ambidentate nature of the thiocyanate anion renders it a very strong nucleophile, capable of reaction directly with the epoxy ring. PGE formulations containing 1-ethyl-3-methylimidazolium thiocyanate appeared to react via the counter ion route in the first instance, whereas those containing 1-ethyl-3- methylimidazolium acetate are thought to proceed via a carbene route. The carbene route is not entirely ruled out for the thiocyanate anion, but this is thought to occur at slightly elevated temperatures (50 °C) when the deprotonation route also becomes active, leading to two reaction pathways competing in the initiation reaction. Additionally, a dealkylation mechanism is suspected for the acetate containing formulations at a temperature of 150 °C where dealkylation products were previously observed via residual gas analysis and NMR spectroscopy [18].

## Figures and Tables

**Table 1:** ^1^H NMR spectral data for equimolar formulation of PGE and 1-ethyl-3-methylimidazolium acetate as a function of storage at room temperature.

	Time (min)	Proton														
		H_6_	H_2_	H_3_	H_A_	H_B_	H_4_	H_C_	H_1_	H_D_	H_E_	H_F_	H_G_	*	H_7_	H_5_
Mult.		s	dd	dd	m	m	q	dd	s	q	m	t	q	s	s	t
Integral	0	2.83	3.46	3.46	9.78	14.47	7.01	4.92	10.27	4.91	4.68	4.83	4.81	2.91	10.52	10.37
	30	3.33	3.95	3.93	8.60	12.86	8.05	4.42	12.04	4.34	4.03	4.17	4.17	2.41	11.97	12.34
	50	2.97	3.93	3.98	8.55	12.78	7.95	4.26	11.54	4.20	3.97	4.09	4.10	3.56	11.62	12.03
	70	0.04	3.63	3.47	8.49	12.38	7.87	4.11	11.62	4.09	3.73	3.91	3.91	0.73	11.37	11.54
	90	0.05	3.67	3.67	8.03	12.07	8.01	3.96	11.80	3.94	3.28	3.65	3.65	0.24	11.42	11.60
Ratio w.r.t. H_A_	0	0.58	0.71	0.71	-	0.99	0.72	1.01	0.70	1.00	0.96	0.99	0.98	0.60	0.72	0.71
	30	0.77	0.92	0.92	-	1.00	0.94	1.03	0.93	1.01	0.94	0.97	0.97	0.56	0.93	0.96
	50	0.69	0.92	0.93	-	1.00	0.93	1.00	0.90	0.98	0.93	0.96	0.96	0.83	0.91	0.94
	70	0.01	0.86	0.82	-	0.97	0.93	0.97	0.91	0.96	0.88	0.92	0.92	0.17	0.89	0.91
	90	0.01	0.91	0.91	-	1.00	1.00	0.99	0.98	0.98	0.82	0.91	0.91	0.06	0.95	0.96

Key: Mult. = multiplicity, s = singlet, dd = doublet of doublets, m = multiplet, q = quartet, t = triplet, - = Not calculated, * = new chemical shift associated with product.

**Table 2:** ^1^H NMR spectral data for equimolar formulation of PGE and 1-ethyl-3-methylimidazolium thiocyanate as a function of storage at room temperature.

	Time (m/d)	Proton														
		H_6_	H_2_	H_3_	H_A_	H_B_	H_4_	H_C_	H_1_	H_D_	H_E_	*	H_F_	H_G_	*	H_5_
Mult. Integral		s	dd	dd	m	m	q	dd	s	q	m	s	t	q	s	t
	0 m	5.56	5.64	5.63	7.54	11.25	15.56		17.13	3.94	3.82	-	3.80	3.79	-	17.17
	20 m	4.55	4.58	4.54	9.58	14.29	14.45		14.16	4.93	4.67	1.30	4.78	4.83	-	14.09
	40 m	4.32	4.61	4.56	9.51	14.22	14.39		14.13	4.95	4.58	1.62	4.75	4.78	-	14.08
	60 m	4.34	4.65	4.60	9.53	14.21	14.37		14.15	4.89	4.61	1.57	4.78	4.76	-	14.10
	90 m	4.37	4.65	4.66	9.57	14.32	14.45		14.31	4.91	4.65	1.39	4.76	4.79	-	14.17
	1 d	4.24	4.47	4.49	9.29	13.95	13.67		13.99	4.37	4.39	1.32	4.21	4.19	3.72	13.58
	8 d	0.17	4.96	5.16	11.12	16.70	13.98		16.85	3.09	2.55	-	1.90	1.79	-	15.04
Ratio w.r.t. H_A_	0 m	1.47	1.50	1.49	-	0.99	1.38		1.51	1.05	1.01	0	1.01	1.01	-	1.52
	20 m	0.95	0.96	0.95	-	0.99	1.00		0.99	1.03	0.97	0.27	1.00	1.01	-	0.98
	40 m	0.91	0.97	0.96	-	1.00	1.01		0.99	1.04	0.96	0.34	1.00	1.01	-	0.99
	60 m	0.91	0.98	0.97	-	0.99	1.01		0.99	1.03	0.97	0.33	1.00	1.00	-	0.99
	90 m	0.91	0.97	0.97	-	1.00	1.01		1.00	1.03	0.97	0.29	0.99	1.00	-	0.99
	1 d	0.91	0.96	0.97	-	1.00	0.98		1.00	0.94	0.95	0.28	0.91	0.90	-	0.97
	8 d	0.03	0.89	0.93	-	1.00	0.84		1.01	0.56	0.46	0	0.34	0.32	-	0.90

Key: Mult. = multiplicity, s = singlet, dd = doublet of doublets, m = multiplet, q = quartet, t = triplet, - = Not calculated, * = new chemical shift associated with product.

## Supplementary Materials

The following are available online at https://www.mdpi.com/2073-4360/11/4/657/s1, Figure S1. 1H NMR spectra of selected ionic liquids; Figure S2. 1H NMR spectra of phenyl glycidyl ether and the diglycidyl ether of bisphenol A; Figure S3. Expected adduct resulting from reaction between 1-methylimidazole and PGE [19]; Figure S4. Recovered imidazole-containing products from the attempted preparation of a 1:1 adduct via reaction between 1-methylimidazole and PGE [19]; Figure S5. Photographs of formulations comprising DGEBA (5g) and 1-ethyl-3-methylimidazolium acetate (0.25g), 1-ethyl-3-methylimidazolium diethyl phosphate (0.25g), 1-ethyl-3-methylimidazolium dicyanamide (0.25g) and 1-ethyl-3-methylimidazolium thiocyanate (0.25g). Samples pictured as a function of storage time at ambient temperature.
