# Supplementary material for: Examining the Influence of Anion Nucleophilicity on the Polymerisation Initiation Mechanism of Phenyl Glycidyl Ether
Source: Polymers (Basel). 2019 Apr 10;11(4):657. doi: 10.3390/polym11040657 (PMC6523680; doi:10.3390/polym11040657)
Supplement: Supplementary file 1 [file polymers-11-00657-s001.pdf]

Supplementary Information

Examining the influence of anion nucleophilicity on the polymerisation initiation mechanism of phenyl glycidyl ether

Fiona C. Binks, Gabriel Cavalli, Michael Henningsen, Brendan J. Howlin, Ian Hamerton*

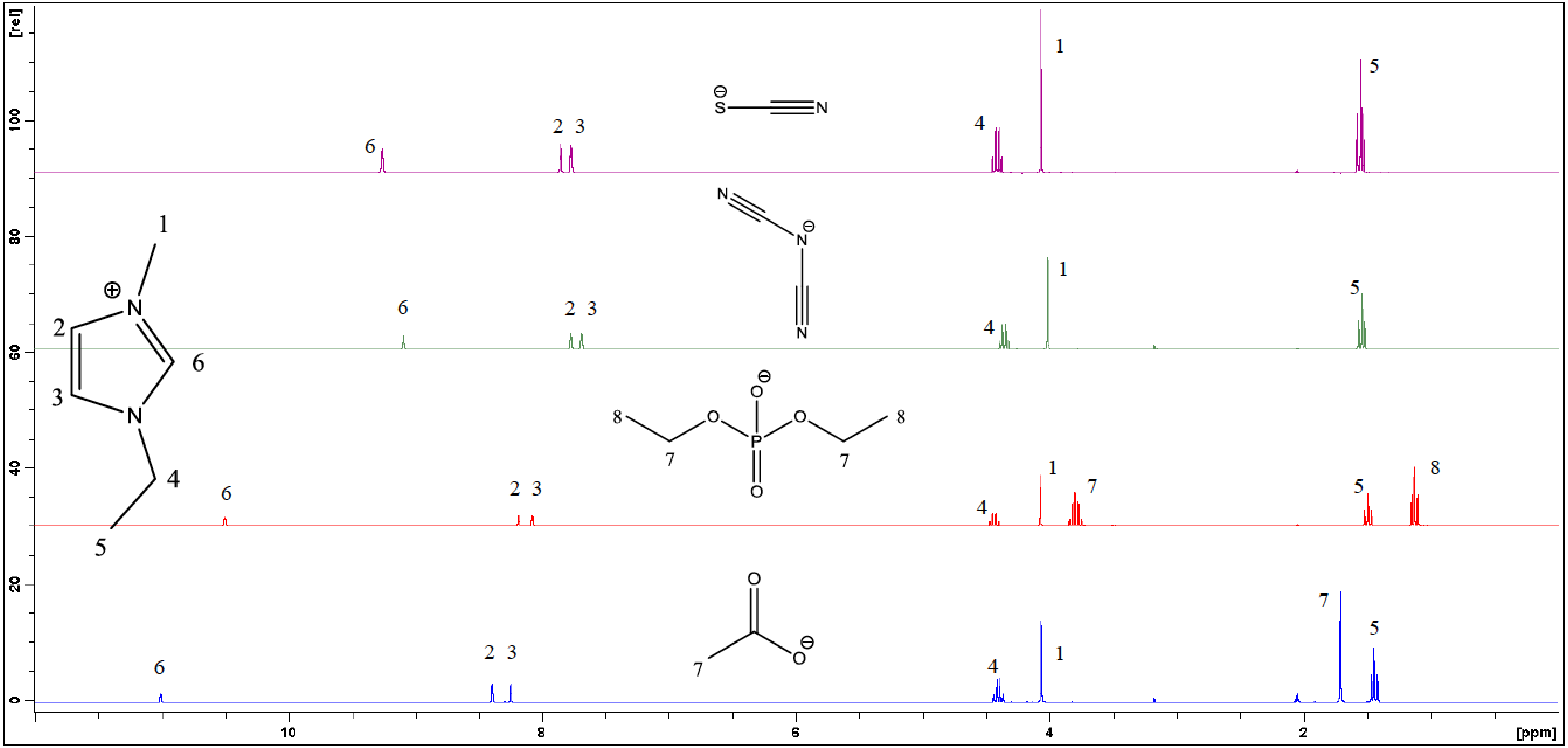

Figure S1. ^{1}H NMR spectra of selected ionic liquids.

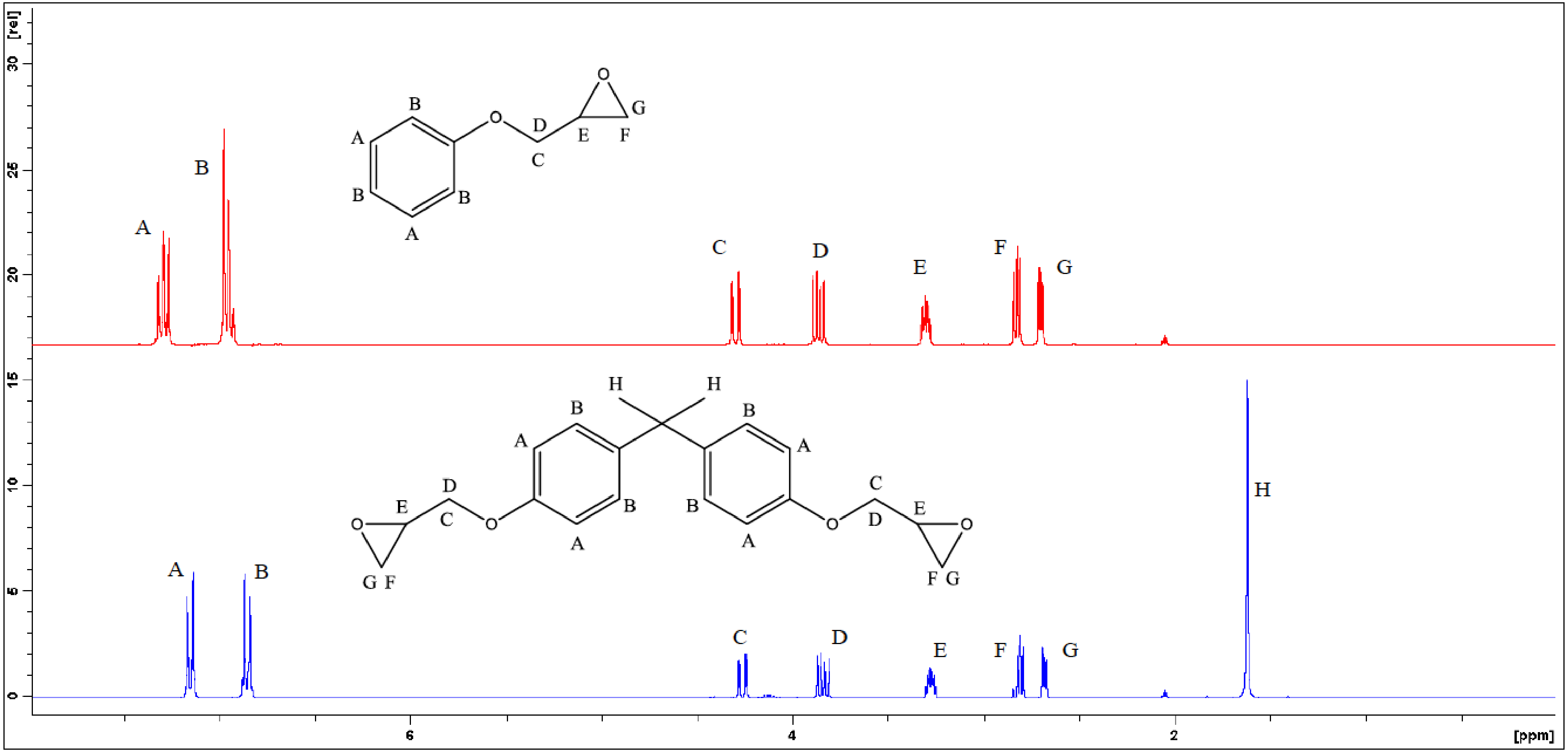

Figure S2. ^{1}H NMR spectra of phenyl glycidyl ether (PGE) and the diglycidyl ether of bisphenol A (DGEBA).

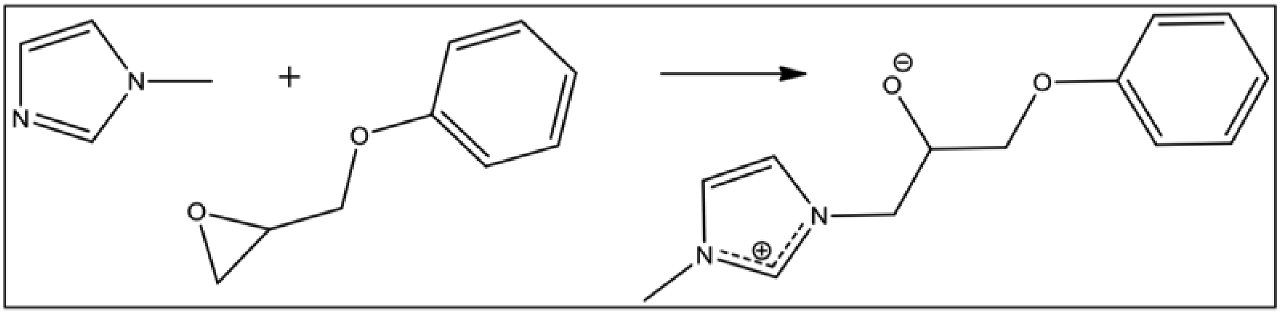

Figure S3. Expected adduct resulting from the reaction between 1-methylimidazole and PGE [19].

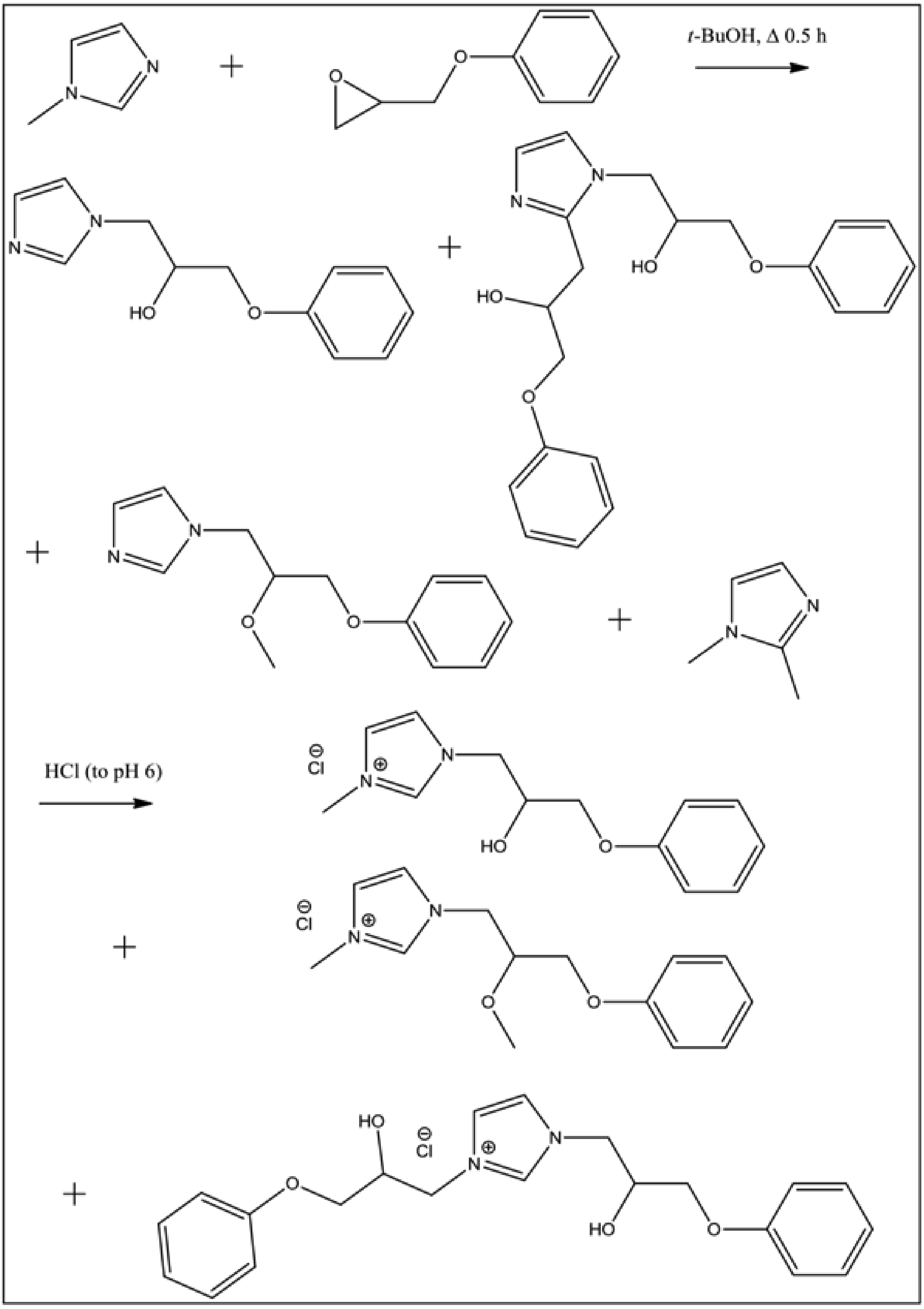

Figure S4. Recovered imidazole-containing products from the attempted preparation of a 1:1 adduct via the reaction between 1-methylimidazole and PGE [19].

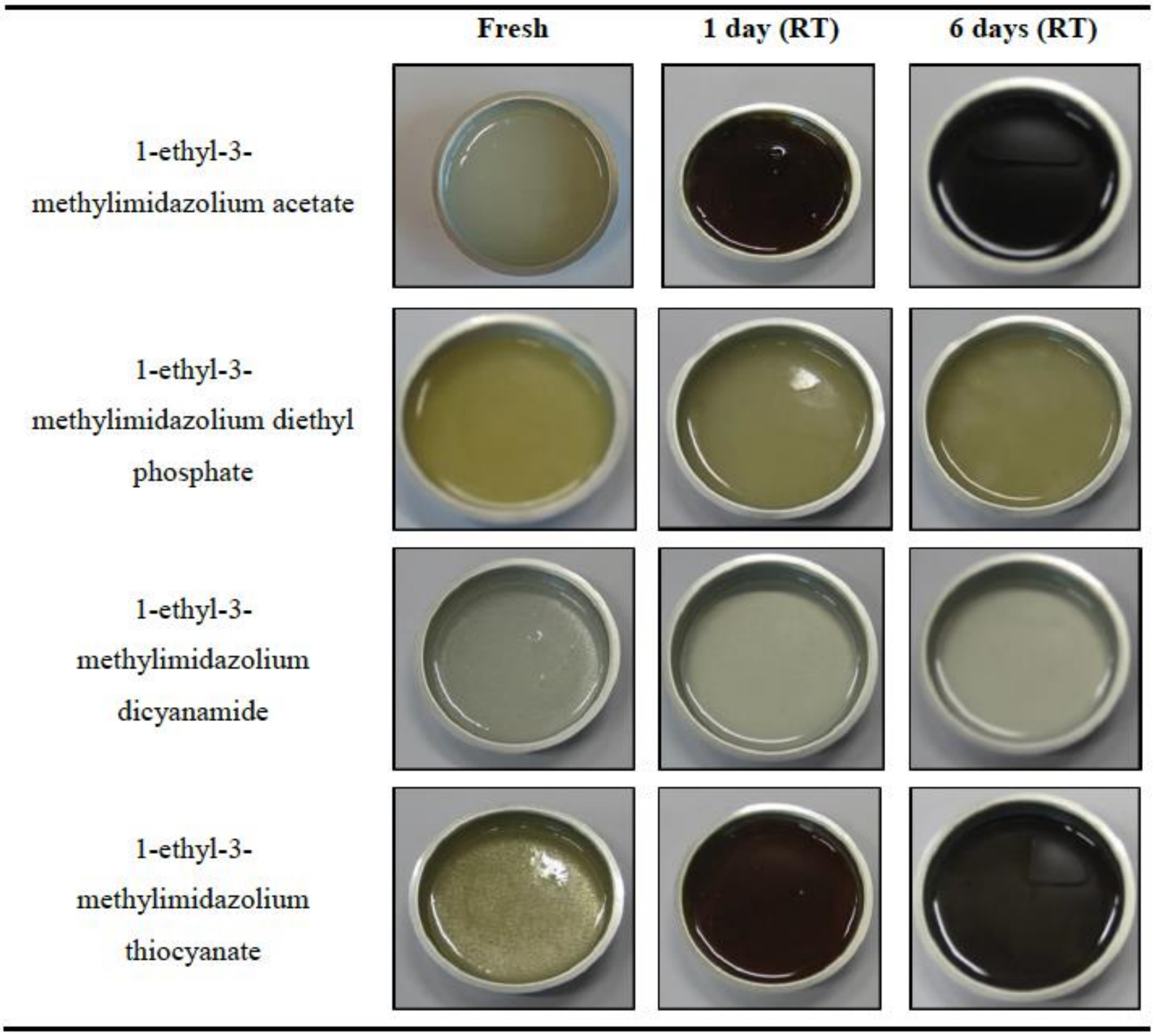

Figure S5. Photographs of the formulations comprising DGEBA (5g) and 1-ethyl-3-methylimidazolium acetate (0.25g), 1-ethyl-3-methylimidazolium diethyl phosphate (0.25g), 1-ethyl-3-methylimidazolium dicyanamide (0.25g), and 1-ethyl-3-methylimidazolium thiocyanate (0.25g). Samples pictured as a function of storage time at ambient temperature.
